# Cardiac ischemia–reperfusion injury under insulin-resistant conditions: SGLT1 but not SGLT2 plays a compensatory protective role in diet-induced obesity

**DOI:** 10.1186/s12933-019-0889-y

**Published:** 2019-07-01

**Authors:** Akira Yoshii, Tomohisa Nagoshi, Yusuke Kashiwagi, Haruka Kimura, Yoshiro Tanaka, Yuhei Oi, Keiichi Ito, Takuya Yoshino, Toshikazu D. Tanaka, Michihiro Yoshimura

**Affiliations:** Division of Cardiology, Department of Internal Medicine, The Jikei University School of Medicine, 3-25-8, Nishi-Shinbashi, Minato-ku, Tokyo, 105-8461 Japan

**Keywords:** SGLT1, Phlorizin, SGLT2-inhibitor, GLUT4, Insulin resistance, Ischemia–reperfusion injury, Glucose uptake

## Abstract

**Background:**

Recent large-scale clinical trials have shown that SGLT2-inhibitors reduce cardiovascular events in diabetic patients. However, the regulation and functional role of cardiac sodium–glucose cotransporter (SGLT1 is the dominant isoform) compared with those of other glucose transporters (insulin-dependent GLUT4 is the major isoform) remain incompletely understood. Given that glucose is an important preferential substrate for myocardial energy metabolism under conditions of ischemia–reperfusion injury (IRI), we hypothesized that SGLT1 contributes to cardioprotection during the acute phase of IRI via enhanced glucose transport, particularly in insulin-resistant phenotypes.

**Methods and results:**

The hearts from mice fed a high-fat diet (HFD) for 12 weeks or a normal-fat diet (NFD) were perfused with either the non-selective SGLT-inhibitor phlorizin or selective SGLT2-inhibitors (tofogliflozin, ipragliflozin, canagliflozin) during IRI using Langendorff model. After ischemia–reperfusion, HFD impaired left ventricular developed pressure (LVDP) recovery compared with the findings in NFD. Although phlorizin-perfusion impaired LVDP recovery in NFD, a further impaired LVDP recovery and a dramatically increased infarct size were observed in HFD with phlorizin-perfusion. Meanwhile, none of the SGLT2-inhibitors significantly affected cardiac function or myocardial injury after ischemia–reperfusion under either diet condition. The plasma membrane expression of GLUT4 was significantly increased after IRI in NFD but was substantially attenuated in HFD, the latter of which was associated with a significant reduction in myocardial glucose uptake. In contrast, SGLT1 expression at the plasma membrane remained constant during IRI, regardless of the diet condition, whereas SGLT2 was not detected in the hearts of any mice. Of note, phlorizin considerably reduced myocardial glucose uptake after IRI, particularly in HFD.

**Conclusions:**

Cardiac SGLT1 but not SGLT2 plays a compensatory protective role during the acute phase of IRI via enhanced glucose uptake, particularly under insulin-resistant conditions, in which IRI-induced GLUT4 upregulation is compromised.

**Electronic supplementary material:**

The online version of this article (10.1186/s12933-019-0889-y) contains supplementary material, which is available to authorized users.

## Background

Although fatty acids are the predominant fuel of energy metabolism in the normal adult heart, glucose is an important preferential substrate under specific pathological conditions, such as ischemia–reperfusion injury (IRI), as it provides greater efficiency for producing high-energy products per oxygen molecule consumed than fatty acids [1–6]. During the acute phase of IRI, under conditions of impaired mitochondrial oxidative phosphorylation, increased glycolysis becomes a major mechanism by which the heart maintains ATP generation and is critical for the cardiomyocyte survival [2]. Therefore, targeting the acceleration of myocardial glucose utilization during the acute phase of IRI is a potential therapeutic strategy for protecting cardiomyocyte and improving the cardiac functional recovery [2, 5, 7, 8]. This approach may become of particular importance under insulin-resistant conditions, such as diabetes mellitus, in which the glucose utilization is further impaired in response to various stimuli, including insulin stimulation as well as ischemic insult.

Glucose utilization is initiated by the uptake of glucose via glucose transporters, which appears to be the rate-limiting step in glycolytic flux in the heart [5, 7, 9]. Glucose transporters are divided into two major families: facilitated glucose transporters (GLUTs) and sodium-coupled active transporters (SGLTs) [6, 7]. Among the 12 subtypes of GLUTs conserved in mammals, GLUT1 and GLUT4 appear to be the major glucose transporters in the heart, and GLUT4 is thought to be the most abundant, accounting for approximately 70% of glucose transporters [6, 9, 10]. GLUT4 resides mainly in the intracellular vesicles under basal conditions and is translocated to the plasma membrane in response to insulin as well as other pathological processes, such as ischemic insult. It was previously reported that GLUT4-mediated enhanced glucose transport represents an essential protective mechanism against IRI and other pathological stresses [2, 8, 9, 11, 12]. However, it was also reported that the cardiac GLUT4 expression is decreased under insulin-resistant conditions, such as diabetes, in association with the reduction in glucose uptake, leading to impaired glucose utilization in the heart [10, 13, 14].

Although the regulation and the functional roles of GLUTs in the heart have been intensively investigated in a variety of in vitro and in vivo models [2, 8, 9, 12–15], less is known about the role and functional significance of SGLTs in the heart, particularly under insulin-resistant conditions. In the heart, SGLT1, not SGLT2, is considered to be the dominant isoform [16–18]. Previous studies showed that SGLT1 is highly expressed in the heart, especially under conditions of ischemia [18, 19], hypertrophy [18, 20], or diabetes [19, 21], all of which were evaluated based on either the mRNA or protein expression from whole heart tissues in the relatively chronic phase. We also recently showed that SGLT1 was highly expressed in the plasma membrane fraction of the human autopsied heart as well as the murine Langendorff perfused heart, although the transmembrane protein expression was not significantly affected, at least during the acute phase of IRI [7]. Subsequently, using a murine Langendorff model, we reported that the non-selective SGLT-inhibitor phlorizin perfusion predisposes the heart to profound IRI, which was associated with a significant decrease in the cardiac tissue ATP content, as a result of a reduction in the glucose uptake as well as the glycolytic flux in the heart during ischemia–reperfusion [7]. These data suggest that cardiac SGLT1 significantly contributes to cardioprotection against IRI by replenishing the ATP stores in ischemic cardiac tissues through promoting glucose utilization. However, the regulation and functional significance of cardiac SGLT1 under insulin-resistant conditions, in which the GLUT4 expression is decreased, remain poorly understood.

Recent studies have shown that chronic excessive actions of SGLT1 actually have a detrimental impact on the heart [20, 22–24]. Considering that SGLT1 was reported to be chronically over-activated in the diabetic heart [19, 21, 25], the question remains whether cardiac SGLT1 detrimentally impacts the diabetic myocardium, or exerts a protective effect against ischemic insult as a compensatory mechanism for compromised GLUT4 under insulin-resistant conditions.

To better understand the role and functional significance of cardiac SGLT1 during IRI under insulin-resistant conditions, we studied the responses of phlorizin-perfused mouse hearts to IRI using high-fat diet (HFD)-induced obese mice.

## Methods

### Animal models

All animal procedures conformed to the National Institutes of Health Guide for the Care and Use of Laboratory Animals and were approved by the Animal Research Committee at the Jikei University School of Medicine (2016-038C4). Male C57BL/6 mice at 8 weeks of age were fed either a HFD—consisting of 60% calories from fat, 20% from protein, and 20% from carbohydrates (D12492; Research Diets, New Brunswick, NJ, USA)—or a matched control normal-fat diet (NFD)—consisting of 10% calories from fat, 20% from protein, and 70% from carbohydrates (D12450J; Research Diets)—for 12 weeks. To investigate the dose effects of SGLT2-inhibitors on myocardial IRI, 10-week-old male ICR mice that had been fed normal chow (CE-2; CLEA Japan, Tokyo, Japan) were used.

### Glucose and insulin tolerance tests

For the glucose tolerance test, mice were fasted for 16 h, anaesthetized (isoflurane, 1%, inhalation), and 1 mg/g body weight of glucose was injected intraperitoneally (i.p.). For the insulin tolerance test, mice were fasted for 2 h, and 1 U/kg body weight of insulin was injected i.p. In both tests, blood samples were collected at baseline and at 15, 30, 60, 90, and 120 min after injection, and plasma glucose concentrations were measured using a commercially available glucose meter (MEDISAFE FIT; Terumo Corporation, Tokyo, Japan).

### Experiments in Langendorff hearts

After 8 to 12 h of fasting, mice were heparinized (1000 IU/kg, i.p.) and anesthetized (pentobarbital, 60 mg/kg, i.p.) in order to eliminate suffering. The heart was then rapidly excised, and the aorta was cannulated onto a Langendorff apparatus, followed by retrograde-perfusion at a constant pressure (80 mmHg) with modified Krebs–Henseleit buffer (11 mM glucose, 118 mM NaCl, 4.7 mM KCl, 2.0 mM CaCl_2_, 1.2 mM MgSO_4_, 1.2 mM KH_2_PO_4_, 25 mM NaHCO_3_, 0.5 mM EDTA), as previously described [7, 11, 26]. A temperature-regulated heart chamber was then placed around the heart in order to keep the perfused heart at a certain temperature. Cardiac hemodynamics was measured using a water-filled balloon catheter introduced into the left ventricle.

### Ischemia–reperfusion model

After a stabilization period of 20 min, the control hearts were perfused for another 10 min before ischemia–reperfusion in order to measure the baseline pre-ischemia cardiac function. Subsequently, global ischemia was applied by eliminating flow followed by reperfusion, as previously described [7]. Where indicated, 10^−4^ mol/L of phlorizin (Sigma-Aldrich, Tokyo, Japan) used as a non-specific SGLT-inhibitor, 5 or 50 µmol/L of tofogliflozin (kindly provided by Sanofi K.K., Tokyo, Japan), 10 µmol/L of ipragliflozin (kindly provided by Astellas Pharma Inc., Ibaraki, Japan), or 15 µmol/L of canagliflozin (kindly provided by Mitsubishi Tanabe Pharma Co., Osaka, Japan), all of which were used as specific SGLT2-inhibitors, was added to the buffer during the pre-ischemia perfusion and reperfusion periods. Phlorizin and SGLT2-inhibitors were dissolved in dimethylsulfoxide (DMSO), and the solvent concentration (0.1% DMSO) was identically maintained in the control group. For the immunoblotting analysis, the individual perfused hearts were snap frozen in liquid nitrogen and stored at − 80 °C prior to protein extraction. Triphenyltetrazolium chloride (TTC) staining was performed to determine the myocardial infarct size using the individual perfused hearts after ischemia–reperfusion, as previously described [7, 11]. Briefly, hearts were sectioned from apex to base into four 2-mm sections. To delineate the infarct, sections were incubated in 5% (wt/vol) TTC in PBS at 37 °C for 20 min. For each section, the area at risk (AAR) and infarct area were measured from enlarged digital micro-graphs. Percent myocardial infarction (%MI) was calculated as the total infarction area divided by the total AAR for that heart.

### Glucose uptake in Langendorff hearts

After 12 h of fasting, the rate of glucose transport in Langendorff perfused hearts was measured by detection of the amount of 2-deoxy-d-glucose (2-DG) using a 2-DG Uptake Measurement kit (Cosmo Bio Co., Ltd., Tokyo, Japan) according to the same protocol as previously described [7].

### Cardiac enzyme measurement

Creatine phosphokinase (CPK) levels were measured in the effluent using an enzymatic activity assay, as previously described [7, 26]. Values were corrected for coronary flow and heart weight [CPK (U/l) × coronary flow (l/min)/heart weight (g) = U/min/g].

### Cardiac muscle fractionation

The preparation and fractionation of plasma membranes from cardiac muscles was performed using Minute Plasma Membrane Protein Isolation Kit (Invent Biotechnologies, Eden Prairie, MN) according to the manufacture’s protocol as previously described [7]. After plasma membrane fractionation, the protein concentrations were determined according to a Bradford assay, and equal amounts of protein were loaded for immunoblotting.

### Immunoblotting

Immunoblotting was performed as previously described [7, 26–28] with rabbit polyclonal anti-SGLT1 (1:200, #H-85; Santa Cruz Biotechnology, CA, USA), anti-GLUT4 (1:1000, #ab33780; Abcam, Cambridge, MA, USA), anti-GLUT1 (1:1000, #12939; Cell Signaling Technology, Tokyo, Japan), or mouse monoclonal anti-α1 Na–K-ATPase (1:1000, #ab7671; Abcam). The signals were detected using chemiluminescence.

### RNA isolation, reverse transcription (RT) and real-time polymerase chain reaction (PCR)

Total RNA was extracted from the frozen tissues using TRIzol reagent (Invitrogen) and a quantitative real-time PCR was performed using a StepOnePlus Real-time PCR System and the StepOne Software program (Applied Biosystems), as described previously [28]. The real-time PCR protocol consisted of one cycle at 95 °C for 20 s followed by 40 cycles at 95 °C for 1 s and 60 °C for 20 s using the primers for Slc5a2 (Mm00453831_m1; Applied Biosystems) and GAPDH (Mn03302249_g1; Applied Biosystems). The transcriptional levels were determined using the ΔΔCt method with normalization to GAPDH.

### Statistical analysis

The data are presented as the mean ± standard error of the mean of at least three independent experiments. Student’s *t*-test was used for the comparison of two data sets. The hemodynamic parameters, %MI determined according to TTC staining and 2-DG uptake were compared using one-way analysis of variance (AONVA) followed by post hoc Bonferroni or Turkey tests. The CPK levels were compared using Kruskal–Wallis test and Mann–Whitney *U* test. A value of P < 0.05 was considered to be significant.

## Results

### Effects of 12-week HFD feeding

**Fig. 1:**
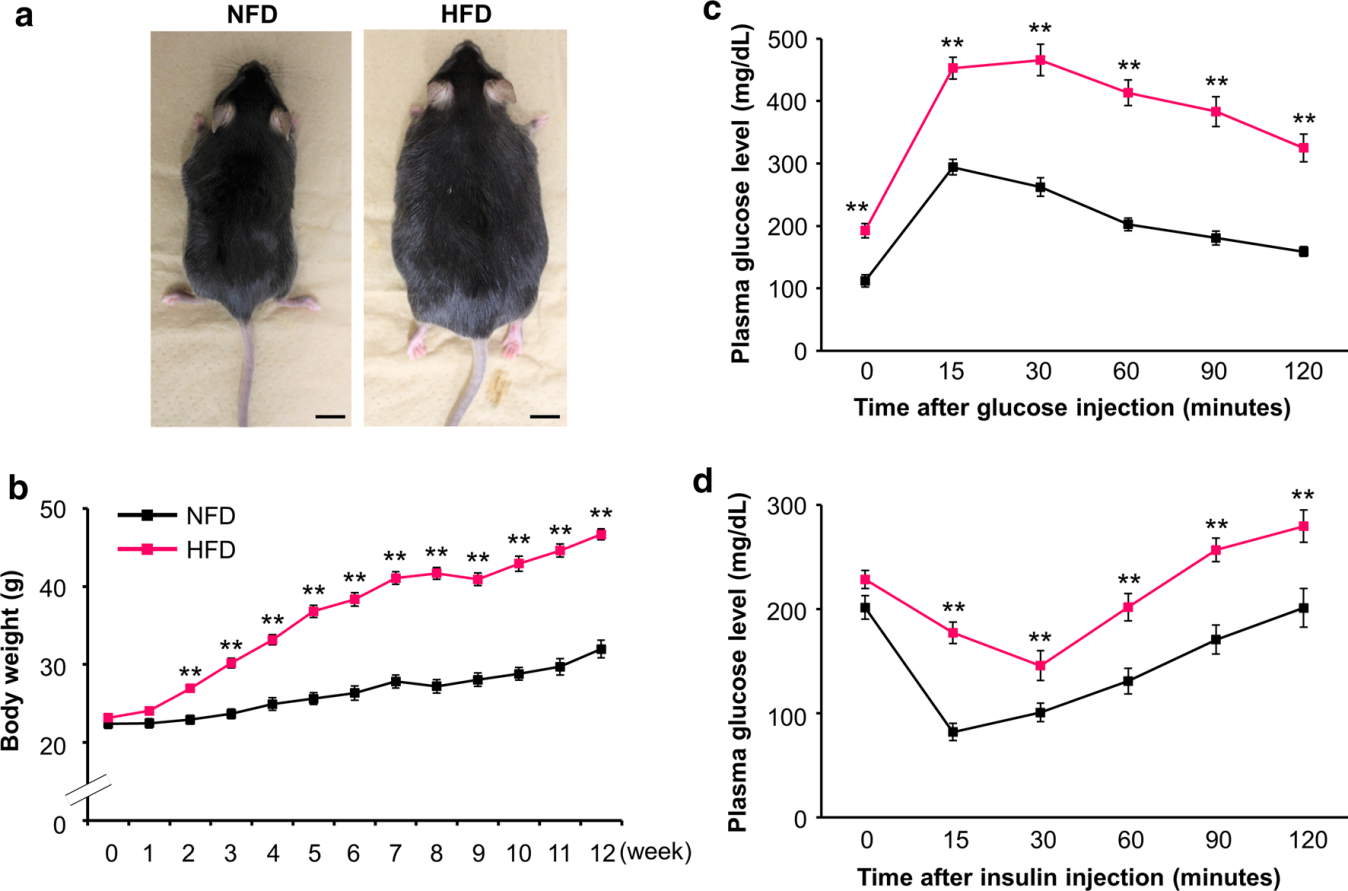
Twelve-week HFD feeding induced obesity, glucose intolerance, and insulin resistance in mice. **a** Appearance of the obese mice after 12 weeks of HFD feeding. Bars = 1 cm. **b** Body weight changes during HFD feeding (n = 6 each). **c** Plasma glucose levels during glucose tolerance tests (NFD; n = 9, HFD; n = 12). **d** Plasma glucose levels during insulin tolerance tests (n = 12 each). **P < 0.01 versus NFD at each time point. *NFD* normal-fat diet, *HFD* high-fat diet

After 12 weeks of HFD feeding, mice developed marked obesity with a 44% increase in body weight compared with NFD mice (Fig. 1a, b). Fasting plasma glucose levels were higher in HFD mice than in NFD mice (Fig. 1c). The glucose tolerance test (Fig. 1c) and insulin tolerance test (Fig. 1d) clearly demonstrated that 12-week HFD feeding induced glucose intolerance and insulin resistance.

### Effects of phlorizin on the cardiac function during IRI in HFD mice

**Fig. 2:**
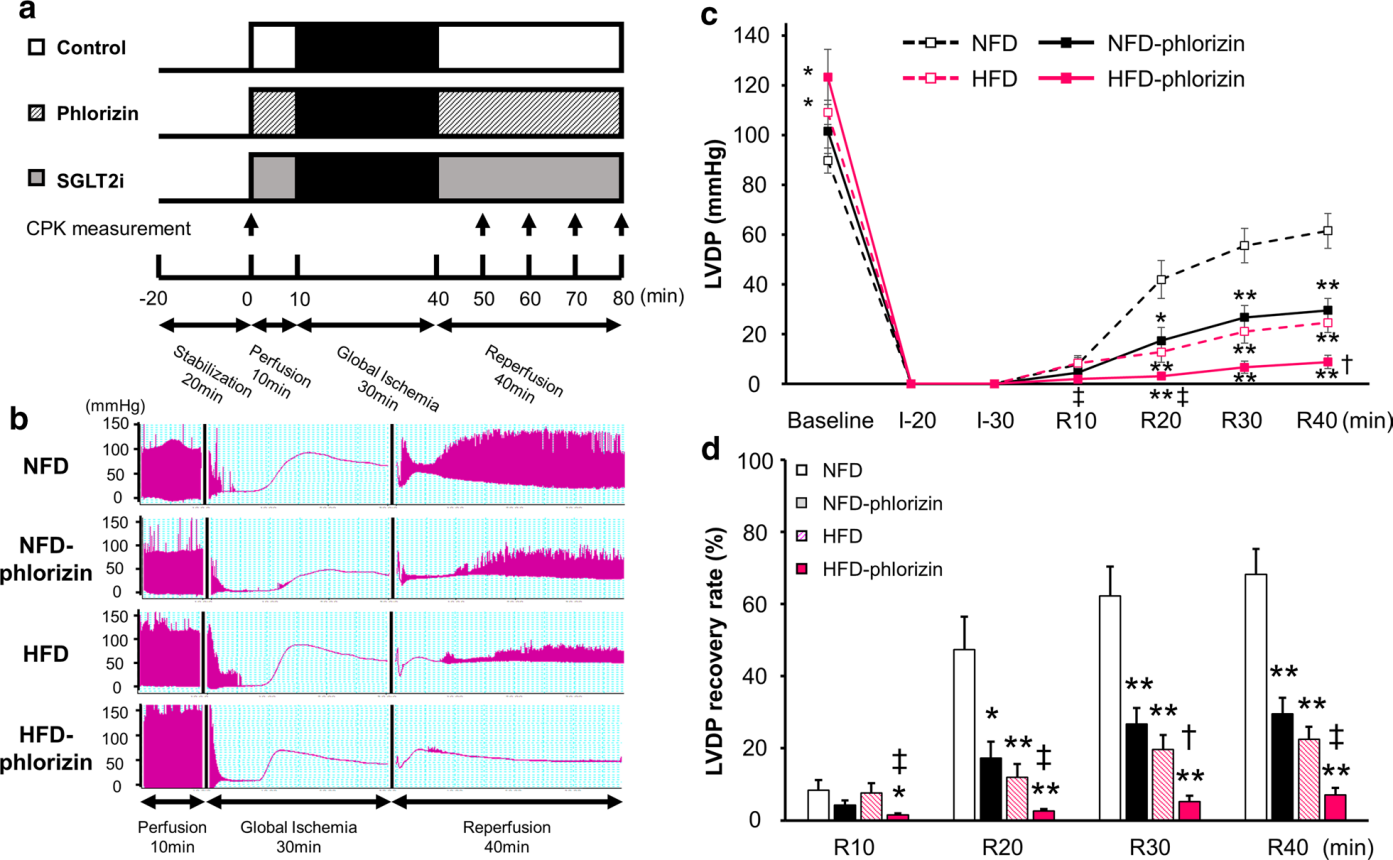
Cardiac functional recovery after ischemia–reperfusion injury with or without phlorizin-perfusion. **a** Experimental protocols indicating the duration and time course of ischemia/reperfusion are shown. The CPK activity released into the perfusate was measured at the point in the protocol indicated by the solid arrows. **b** Representative tracing of LVDP during ischemia–reperfusion with or without phlorizin-perfusion. LVDP profiles (**c**) and LVDP recovery (percent of baseline) (**d**) measured at the indicated time points during ischemia–reperfusion in NFD (open black square; n = 6), NFD phlorizin-perfused (filled black square; n = 6), HFD (open pink square; n = 8), and HFD phlorizin-perfused (filled pink square; n = 6) hearts are shown. *P < 0.05 and **P < 0.01 versus the NFD group at each time point; ^†^P < 0.05 and ^‡^P < 0.01 versus the HFD group at each time point. *SGLT2i* SGLT2-inhibitors, *min* minutes, *LVDP* left ventricular developed pressure, *I* ischemia, *R* reperfusion

The experimental protocols of ex vivo Langendorff ischemia–reperfusion (Fig. 2a) and the representative tracing of left ventricular developed pressure (LVDP) during IRI (Fig. 2b) are shown. The baseline cardiac function measured at the end of the 10-min pre-ischemia perfusion period indicated that HFD mice had increased contractile activity, consistent with the previous studies (Table 1) [29, 30]. Phlorizin-perfusion did not significantly affect the baseline cardiac function in hearts of either NFD or HFD mice. After 30-min global ischemia followed by 40-min reperfusion, HFD per se significantly reduced LVDP recovery (22.5% ± 3.5% versus 68.3% ± 7.1% recovery from baseline, P < 0.01, Fig. 2c, d) and the rate pressure product (RPP), calculated as LVDP × heart rate, in order to consider the impact of the heart rate on the cardiac function (6733 ± 1271 versus 20,610 ± 4716 mmHg bpm at the end of IRI, P < 0.01, Additional file 1: Fig. S1A) in association with an increase in left ventricular end-diastolic pressure (LVEDP) (Additional file 1: Fig. S1B) and the significant impairment of the maximum rate of contraction (+dp/dt_max_) and maximum rate of relaxation (−dp/dt_min_) (Additional file 1: Fig. S1C) compared with those values in NFD hearts. Although phlorizin-perfusion also impaired the cardiac functional recovery in NFD hearts (LVDP recovery rate: 29.5% ± 4.5%, P < 0.01, RPP: 9102 ± 867 mmHg bpm, P < 0.05), consistent with our previous study [7], further dramatically impaired cardiac functional recovery was observed in HFD hearts with phlorizin-perfusion (LVDP recovery rate: 7.1% ± 1.9%, RPP 2146 ± 601 mmHg bpm, P < 0.01 versus HFD without phlorizin-perfusion). These data suggest that SGLT1 is vital for the cardiac functional recovery after ischemia–reperfusion, particularly under insulin-resistant conditions.

**Table 1:** Baseline cardiac function of ex vivo perfused hearts with or without phlorizin-perfusion

	NFD (n = 6)	NFD-phlorizin (n = 6)	HFD (n = 8)	HFD-phlorizin (n = 8)
LVSP, mmHg	98.9 ± 5.1	112 ± 8.5	119 ± 4.9*	133 ± 11*
LVEDP, mmHg	9.2 ± 0.4	10 ± 0.8	9.6 ± 0.2	9.4 ± 0.5
LVDP, mmHg	89.8 ± 5.1	102 ± 8.9	109 ± 5.0*	123 ± 11*
+dp/dt, mmHg/s	2742 ± 247	3277 ± 308	3478 ± 162	4098 ± 463
−dp/dt, mmHg/s	− 2308 ± 200	− 2450 ± 277	− 2814 ± 177	− 3380 ± 518
HR, bpm	272 ± 44	306 ± 35	266 ± 27	284 ± 22
RPP, mmHg bpm	25,429 ± 5192	31,251 ± 4897	28,650 ± 2714	35,970 ± 5245
Coronary flow, mL/min	3.30 ± 0.47	2.93 ± 0.36	3.19 ± 0.32	3.58 ± 0.38

*NFD* normal fat diet, *HFD* high fat diet, *LVSP* left ventricular systolic pressure, *LVEDP* left ventricular end-diastolic pressure, *LVDP* left ventricular developed pressure, *HR* heart rate, *RPP* rate pressure product

*P < 0.05 versus NFD group

### Cardiac injury after ischemia–reperfusion

**Fig. 3:**
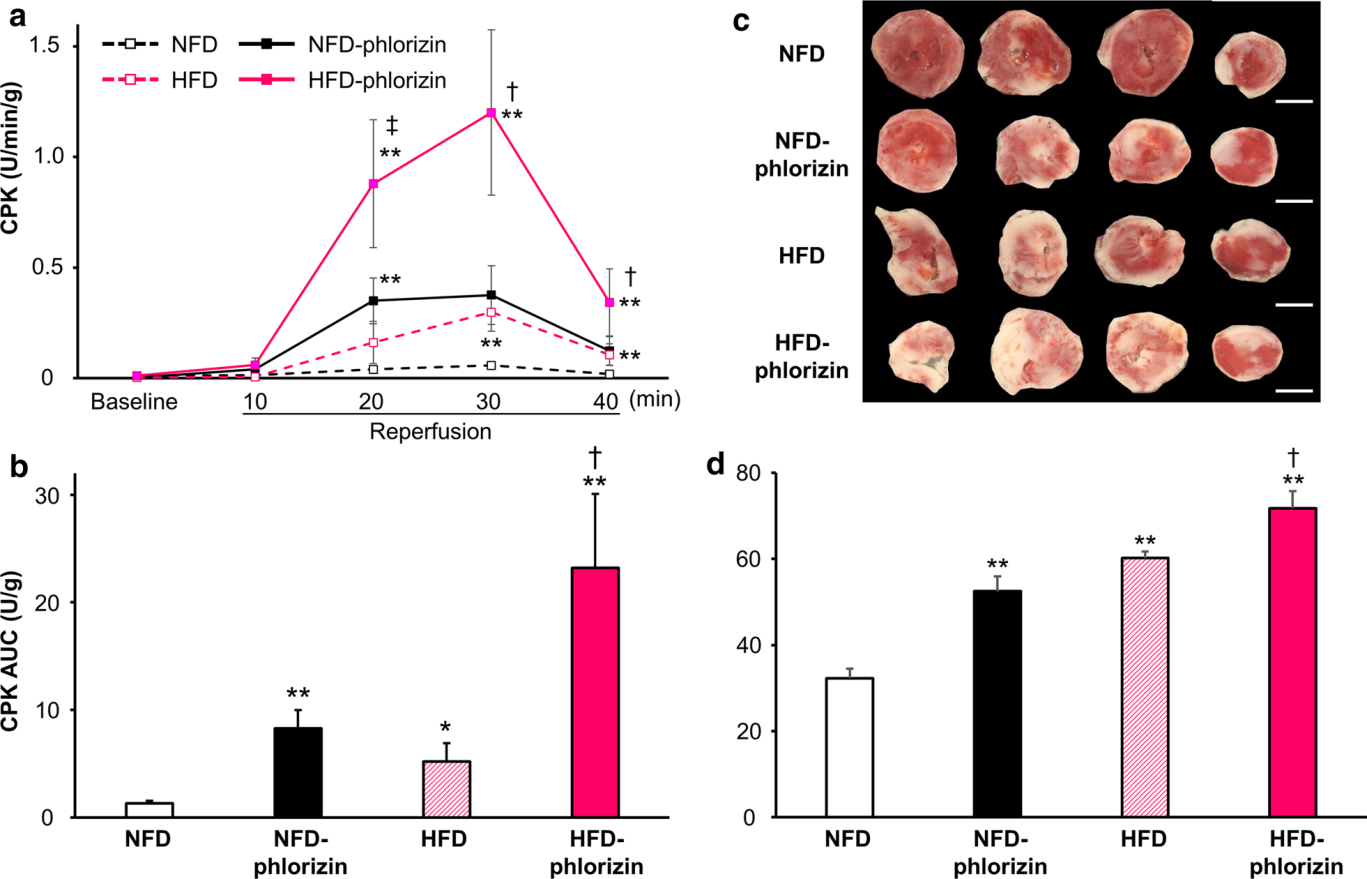
Myocardial injury after ischemia–reperfusion with or without phlorizin-perfusion. **a** CPK profiles in the effluent collected during the reperfusion period. **b** The area under the curve (AUC) was calculated from the CPK profile shown in **a** (NFD; n = 11, NFD with phlorizin-perfusion; n = 7, HFD; n = 10, HFD with phlorizin-perfusion; n = 4). *P < 0.05 and **P < 0.01 versus the NFD group at each time point; ^†^P < 0.05 and ^‡^P < 0.01 versus the HFD group at each time point. **c** Micrograph showing representative TTC staining of cardiac sections obtained from NFD, NFD with phlorizin-perfusion, HFD, and HFD with phlorizin-perfusion groups after ischemia–reperfusion. Bars = 3 mm. **d** Effects on the quantitated cumulative infarct area size in NFD (n = 9), NFD phlorizin-perfused (n = 6), HFD (n = 5), and HFD phlorizin-perfused (n = 4) hearts. **P < 0.01 versus the NFD group; ^†^P < 0.05 versus the HFD group. *%MI* myocardial infarct area/ventricular area

The activity of CPK released into the perfusate during reperfusion was measured as an index of myocardial injury (Fig. 3a), and the total amount of CPK released during the entire reperfusion period, as indicated by the CPK area under the curve (AUC) for the CPK level, was also shown (Fig. 3b) [7, 11]. During the baseline pre-ischemia perfusion period, no CPK activity was detectable. After IRI, the CPK activity was significantly increased in HFD hearts compared with NFD hearts (CPK-AUC: 5.2 ± 1.7 versus 1.3 ± 0.2 U/g, P < 0.05). Although phlorizin-perfusion also increased CPK release in NFD hearts (8.3 ± 1.7 U/g, P < 0.01), consistent with our previous study [7], further dramatically increased CPK release was observed in HFD hearts with phlorizin-perfusion (23.2 ± 6.9 U/g, P < 0.05 versus HFD without phlorizin-perfusion). These findings correlated with larger infarcts in NFD hearts with phlorizin-perfusion and HFD hearts (52.5% ± 3.5% [P < 0.01] and 60.2% ± 1.5% [P < 0.01], versus 32.3% ± 2.2% [NFD], respectively), which was particularly striking in HFD hearts with phlorizin-perfusion (71.8% ± 4.0%, P < 0.05 versus HFD hearts without phlorizin-perfusion), compared with those noted in the NFD hearts (Fig. 3c, d).

### Direct effects of selective SGLT2-inhibitors on either NFD or HFD hearts during IRI

Increasing attention has been focused on SGLT2-inhibitors as novel anti-diabetic agents that reduce cardiovascular events, as shown in recent large clinical trials [31–33]. We therefore next investigated the direct effects of selective SGLT2-inhibitors on the cardiac functional recovery and myocardial injury after ischemia–reperfusion in both HFD and NFD hearts.

**Fig. 4:**
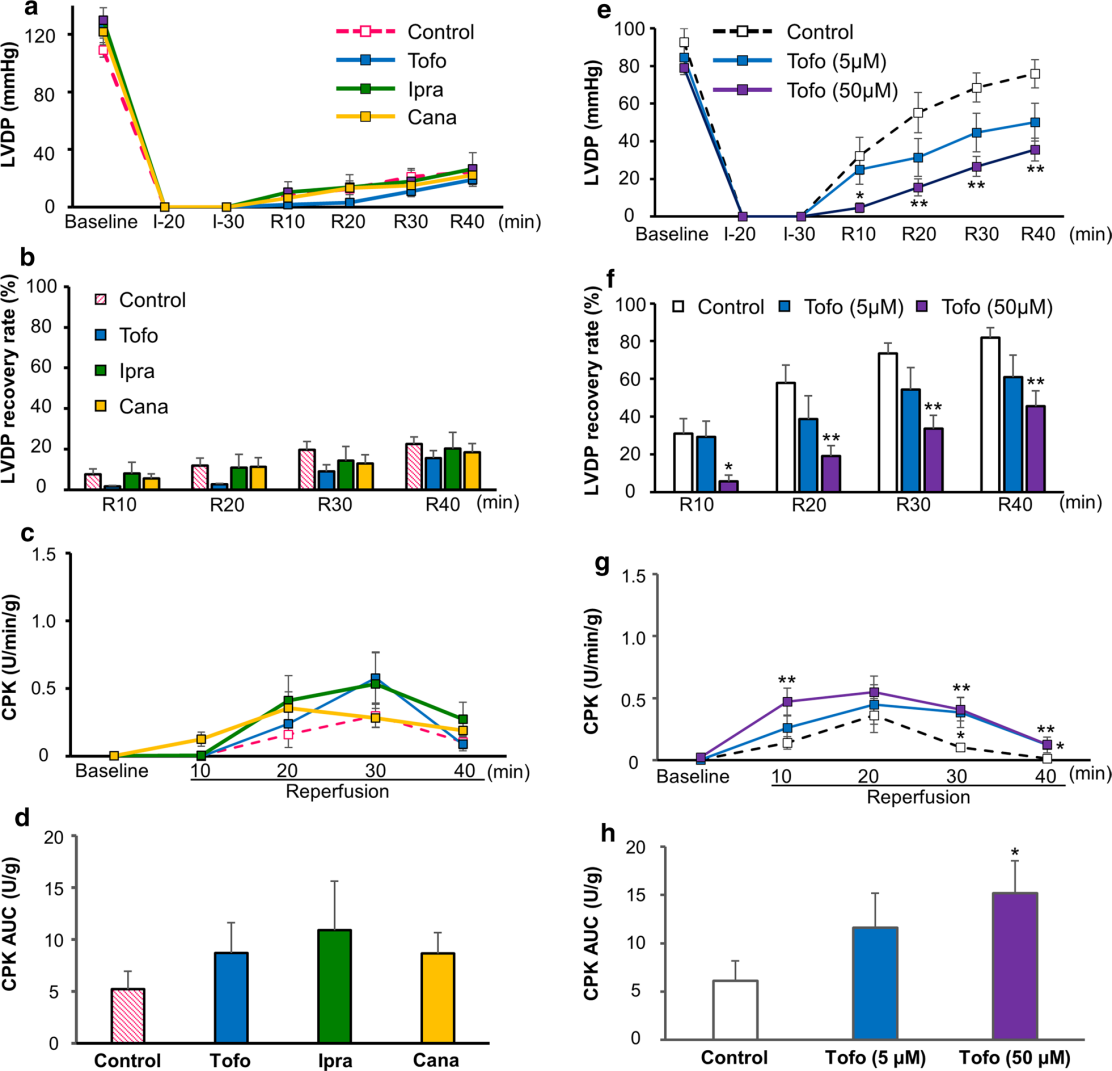
Cardiac functional recovery and myocardial injury after ischemia–reperfusion injury with or without SGLT2-inhibitor perfusion. LVDP profiles and myocardial injury in HFD (**a**–**d**) and NFD (**e**–**h**) mice are shown. LVDP profiles (**a**) and LVDP recovery (percent of baseline) (**b**) measured at the indicated time points during ischemia–reperfusion in the control (open pink square; n = 8, as shown in Fig. 2c, d), 5 µM tofogliflozin-perfused (blue square; n = 5), 10 µM ipragliflozin-perfused (green square; n = 6), and 15 µM canagliflozin-perfused (yellow square; n = 8) hearts are shown. CPK profiles in the effluent collected during the reperfusion period (**c**) and the area under the curve (AUC) (**d**) calculated from the CPK profile (**c**) are shown (control: n = 5, as shown in Fig. 3a, b; tofogliflozin perfusion: n = 5; ipragliflozin perfusion: n = 7; canagliflozin perfusion: n = 7). **P < 0.01 versus the HFD group at each time point. LVDP profiles (**e**) and LVDP recovery (percent of baseline) (**f**) measured at the indicated time points during ischemia–reperfusion in the control (open black square; n = 10), 5 µM tofogliflozin-perfused (blue square; n = 8), and 50 µM tofogliflozin-perfused (purple square; n = 8) hearts are shown. CPK profiles in the effluent collected during the reperfusion period (**g**) and the AUC (**h**) calculated from the CPK profile (**g**) are shown (control: n = 12; tofogliflozin [5 µM]-perfusion: n = 7; tofogliflozin [50 µM]-perfusion: n = 7). *P < 0.05 and **P < 0.01 versus control at each time point. *Tofo* tofogliflozin, *Ipra* ipragliflozin, *Cana* canagliflozin

No SGLT2 mRNA expression was detected even in HFD mouse hearts (Additional file 1: Fig. S2). Each SGLT2-inhibitor—namely 5 µM of tofogliflozin, 10 µM of ipragliflozin, or 15 µM of canagliflozin—was perfused according to the protocol of Langendorff ischemia–reperfusion using HFD hearts (Fig. 2a), and no significant effects of these SGLT2-inhibitors on the baseline cardiac function were noted (Additional file 1: Table S1). After IRI, none of the SGLT2-inhibitors significantly affected the cardiac function, including LVDP recovery after IRI, or CPK release in HFD mice (Fig. 4a–d).

To simply assess the dose effects of SGLT2-inhibitors, either 5 or 50 µM of tofogliflozin was perfused during 20-min global ischemia followed by 40-min reperfusion in ICR mice fed normal chow. The baseline cardiac function was not affected by either dose of tofogliflozin (Additional file 1: Table S2). However, while 5 µM of tofogliflozin did not affect the cardiac function or CPK release during IRI, 50 µM of tofogliflozin significantly reduced LVDP recovery in association with an increased CPK release after IRI (Fig. 4e–h), similar to the effects observed under phlorizin-perfusion [7]. Taken together, these findings indicated no harmful effects of selective SGLT2-inhibitors during IRI, even in HFD hearts, although detrimental effects were noted at supra-pharmacological doses, possibly through non-selective SGLT1 inhibition.

### The expression of glucose transporters in HFD hearts during IRI

**Fig. 5:**
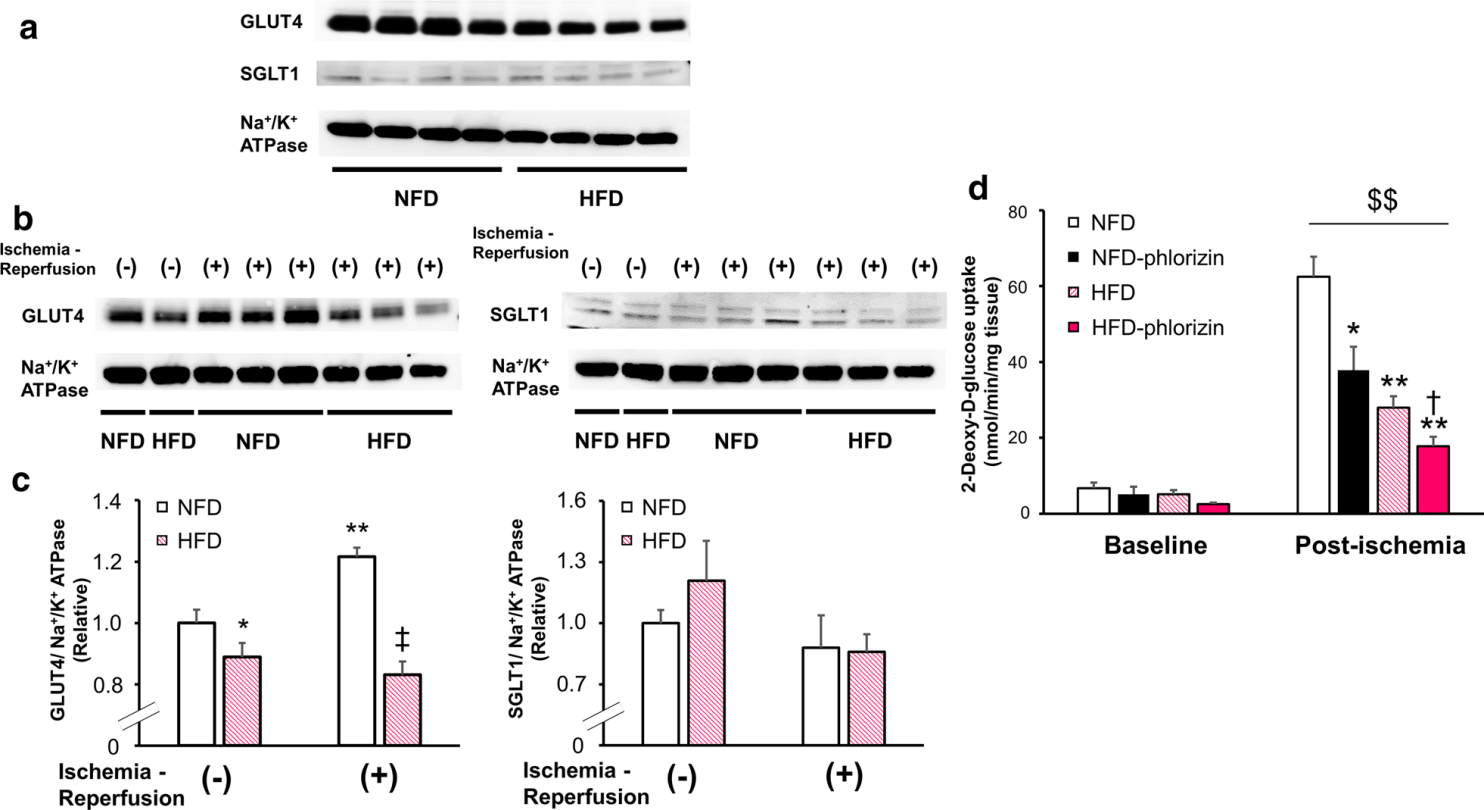
The expression of glucose transporters and the glucose uptake in myocardium during ischemia–reperfusion. Representative immunoblots of GLUT4 and SGLT1 in the plasma membrane fraction from the murine perfused hearts at baseline measured at the end of 10-min pre-ischemia perfusion (**a**) and before and after IRI (**b**) are shown. **c** Densitometric quantitation normalized to the level of either the GLUT4 or SGLT1 expression in NFD hearts before IRI are shown ([GLUT4] NFD or HFD without IRI: n = 8 each, NFD or HFD with IRI: n = 6 each; [SGLT1] NFD or HFD without IRI: n = 9 each, NFD or HFD with IRI: n = 7 or n = 6, respectively). *P < 0.05, **P < 0.01 versus NFD hearts before IRI; ^‡^P < 0.01 versus NFD hearts after IRI. In both **a** and **b**, immunoblots of Na^+^/K^+^ ATPase from the same membrane are shown as a loading control for the membrane fraction. **d** Glucose uptake in NFD (open black square), NFD phlorizin-perfused (filled black square), HFD (open pink square), and HFD phlorizin-perfused (filled pink square) hearts under the pre-ischemic baseline conditions and post-ischemic condition measured after 20-min reperfusion following 30-min global ischemia (baseline/post-ischemic condition, NFD: n = 4/n = 5, NFD with phlorizin-perfusion: n = 3/n = 5, HFD: n = 7/n = 7, and HFD with phlorizin-perfusion: n = 3/n = 7) *P < 0.05 and **P < 0.01 versus NFD hearts under post-ischemic conditions, ^†^P < 0.05 versus HFD hearts under post-ischemic conditions, ^$$^P < 0.01 versus corresponding controls at baseline

In order to clarify the mechanisms underlying these detrimental effects of SGLT1 inhibition during IRI, particularly in HFD hearts, we next examined the dynamics of glucose transporters in these murine hearts. Immunoblotting with plasma membrane fractionation revealed that the GLUT4 expression was significantly reduced in HFD hearts compared with NFD hearts at baseline (Fig. 5a, c). The GLUT4 expression at the plasma membrane was significantly increased after IRI in NFD hearts, while the response of GLUT4 to IRI was substantially attenuated in HFD hearts (Fig. 5b, c). In contrast, the SGLT1 expression at the plasma membrane remained constant during IRI, regardless of diet conditions (Fig. 5a–c). The GLUT1 expression was also equivalent during IRI under both diet conditions (Additional file 1: Fig. S3). Likewise, the regulation of the expression of those glucose transporters was not affected by phlorizin-perfusion (Additional file 1: Fig. S4), indicating that SGLT1 inhibition negligibly influences the regulation of GLUT4 and GLUT1 expression in NFD and HFD hearts, both at baseline and after IRI. In addition, these data may rule out the possibility of the non-specific inhibitory effects of phlorizin on other glucose transporters, at least in the present experimental model.

### Glucose utilization in the hearts during IRI

To test the functional significance of the contrasting changes in the plasma membrane expression of glucose transporters, we measured the myocardial glucose uptake (one of the major determinants of myocardial glucose utilization) before and after IRI [7]. At baseline, there were no significant between-group differences in the glucose uptake rate (Fig. 5d). In contrast, during ischemia–reperfusion, the cardiac glucose uptake was dramatically enhanced by the ischemic insult in NFD hearts, which was significantly attenuated in HFD hearts. This was thought to be mainly due to the blunted GLUT4 reaction to IRI. Of note, phlorizin considerably reduced the glucose uptake after IRI, particularly in HFD hearts compared to the other subjects (Fig. 5d). These data suggest that SGLT1 plays a critical role in myocardial glucose utilization during IRI, particularly under insulin-resistant conditions.

## Discussion

In the present study using diet-induced obesity model, we showed that the inhibition of cardiac SGLT1 by phlorizin during the acute phase of IRI led to an impaired cardiac functional recovery and increased myocardial injury, which were associated with significant reductions in the myocardial glucose uptake enhanced by IRI. The novel findings of the present study are as follows: (i) the detrimental influence of phlorizin during IRI described above was more striking in subjects with diet-induced obesity than in normal subjects. (ii) This mechanistic connection was demonstrated by the blunted response of GLUT4 upregulation to IRI in contrast to the constant expression of SGLT1 in diet-induced obesity mouse hearts. (iii) In contrast to phlorizin, selective SGLT2-inhibitors did not significantly affect the cardiac functional recovery or myocardial injury after ischemia–reperfusion, even in diet-induced obesity mouse hearts, in which the SGLT2 expression was not detected. These data indicate the increasing reliance on SGLT1 for cardiac functional recovery and energy metabolism during IRI in insulin-resistant diabetic phenotypes, in which IRI-induced GLUT4 upregulation is compromised.

Various mechanisms underlying the detrimental influence of hyperglycemia under diabetic and/or insulin-resistant conditions on the myocardial function, structure, and energy metabolism have been intensively investigated [34–37]. One key point to note in the present isolated heart perfusion study is that the detrimental effects of HFD on these parameters became evident after ischemia–reperfusion, not being noted at baseline before ischemia. In this context, one possible mechanism is the compromised GLUT4 translocation. Previous studies showed that GLUT4 is an important mediator of enhanced glycolysis and maintaining ATP concentration under various pathological conditions, including IRI [2, 8, 9, 12]. However, significant decreases in the sarcolemmal GLUT4 expression were observed in insulin-resistant obese mouse hearts [13, 14], consistent with the present findings. Furthermore, we obtained the new finding that the GLUT4 upregulation in response to IRI insult was significantly attenuated in HFD mice, in association with the decreased myocardial glucose uptake after IRI. The impaired GLUT4 expression and translocation in diabetic subjects may involve not only disturbance of insulin signaling but also increased membrane cholesterol, reductions in membrane fluidity, and disruption of caveolae and caveolin-3 [34]. These findings may, at least in part, account for the impaired cardiac functional recovery and increased myocardial injury after ischemia–reperfusion in HFD hearts.

The same logic can be applied here: The present finding that an increased myocardial glucose uptake in response to IRI was blocked by phlorizin may be the main mechanism underlying the poor functional recovery and increased myocardial injury after ischemia–reperfusion. This aligns with our previous findings showing that cardiac SGLT1 is involved in an important protective mechanism against IRI by replenishing ATP stores in ischemic cardiac tissues via enhanced glucose utilization [7]. Consistent with a series of our studies, Connelly et al. showed that oral administration of a dual SGLT1/2-inhibitor led to an impaired cardiac function after myocardial infarction in a rat model with left anterior descending coronary artery ligation, while a selective SGLT2-inhibitor had no significant effect on this condition [38]. These data indicate that cardiac SGLT1 exerts protective effects against myocardial ischemia even in vivo, although they used a non-diabetic model rather than HFD-induced diabetic subjects. Furthermore, Kanwal et al. showed that SGLT1 inhibition by phlorizin abrogated the beneficial effect of ischemic preconditioning against IRI [39]. However, while cardiac SGLT1 does indeed exert favorable effects during the acute phase of IRI, its chronic excessive activation has been reported to have unfavorable effects [20–23, 25], thus suggesting that the time course of SGLT activation is critical for eliciting cardioprotective effects [7]. Indeed, Li et al. very recently reported that the RNAi-mediated knockdown of SGLT1 in cardiomyocytes is protective against IRI by reducing oxidative stress [24], although they found a similar exacerbation of IRI when using phlorizin. There may also be some compensatory mechanisms activated in those mouse hearts by the permanent knock-down of cardiac SGLT1 from birth. Considering that the SGLT1 expression is chronically increased under diabetic conditions [19, 21], resulting in detrimental effects [21, 25, 40], it is important to attain the effects of SGLT1 on the cardiac function as well as energy metabolism during acute phase of IRI, particularly in insulin-resistant diabetic models. We found in the present study that, under insulin-resistant conditions, SGLT1 isoform may play a compensatory protective role during the loss of GLUT4-mediated glucose uptake, rather than provides detrimental impacts on the diabetic myocardium [21, 25], at least during the acute phase of IRI.

Despite the absence of changes in the SGLT1 expression, even under insulin-resistant conditions, our results still indicate the possible role of SGLT1 activation in the cardiomyocytes (in addition to its translocation/internalization-mediated expression), considering that phlorizin exerted substantial effects on the cardiac functional recovery and injury as well as the myocardial glucose uptake after ischemia–reperfusion in the HFD-induced obese mice. However, the precise regulatory mechanisms by which SGLT1 develops tolerance to IRI under insulin-resistant conditions remain unclear. The cardiac SGLT1 is thought to be activated by various factors, such as insulin, AMP-activated protein kinase (AMPK), persistent hyperglycemia, as well as ischemia–reperfusion injury per se [18–21, 24, 40], all of which can also activate GLUT4. And thus, a pathway specific for cardiac SGLT1 activation has not yet been identified. Hypoxia-inducible factor 1α (HIF-1α), which is increased under diabetic as well as hypoxic conditions, is a major regulator of the GLUT1 expression (although the GLUT1 expression was not significantly affected in the present study; Additional file 1: Figs. S3 and S4). As such, it may play a potential role in regulating the SGLT1 activity in the heart, although it has recently been shown to diminish the SGLT1 and SGLT2 expression in kidneys [41], which is the opposite of what we might expect based on the findings of the present study. Future investigations should clarify the mechanisms underlying the regulation of the SGLT1 expression/activation in the cardiomyocytes in greater detail and identify the specific regulators and/or agents (namely, SGLT1-specific agonists) that transiently induce the expression/activation of cardiac SGLT1 during IRI, which may lead to potential therapeutic applications for the acute phase of IRI.

A recent series of large-scale clinical trials has shown that SGLT2-inhibitors have profound benefits on reducing the risk of cardiovascular events in patients with type 2 diabetes [31–33]. In this context, assessing the direct effects of SGLT2-inhibitors on the heart in the current experimental model has important clinical implications. We did not detect any SGLT2 expression, even in HFD mouse hearts, which explains that tofogliflozin, ipragliflozin, and canagliflozin, all of which are selective SGLT2-inhibitors, did not affect the cardiac functional recovery or myocardial injury after IRI. To confirm the safety and efficacy of these recently developed anti-diabetic agents, the dosage used in the current study was almost fivefold higher than the blood concentrations after a single oral dose of each SGLT2-inhibitor in clinical use. There is growing evidence showing the potential cardioprotective mechanisms of SGLT2-inhibitors, largely mediated through (as systemic effects) decreasing the insulin resistance by reducing the body fat mass, modulating natriuretic peptides, reducing arterial stiffness, modulating sympathetic tone, exerting renoprotective effects, and (as direct cardiac effects) reducing inflammation, oxidative stress, apoptosis, fibrosis, inhibiting sodium–hydrogen exchanger (NHE) [42–44]. The neutral effect of SGLT2-inhibitors in the present study can be partly explained by the fact that the current experimental model is an ex vivo isolated heart perfusion model, which eliminates the systemic effects of SGLT2-inhibitors. Meanwhile, a series of recent studies by Zuurbier et al. clearly showed that selective SGLT2-inhibitors directly inhibit cardiac NHE [45–47], although empagliflozin did not significantly affect the cardiac functional recovery or myocardial injury after ischemia–reperfusion, which is consistent with our findings; however, the authors did not examine any models of diet-induced obesity [45]. In addition, Lee et al. reported that dapagliflozin attenuated cardiac fibrosis by regulating macrophage polarization [48], although most macrophage and other blood cells were washed out in our present study model of Langendorff. Intriguingly, they indicated that dapagliflozin induced compensatory activation of SGLT1 as a cardio-protective mechanism, leading to a reduction in oxidative stress. Based on these previous findings, it is possible that, in our current study, selective SGLT2-inhibitors exerted direct cardioprotective effects, although further studies will be needed in order to investigate the effects of those pharmacological agents on cardiac NHE under diabetic insulin-resistant conditions as well as on cardiac fibrosis through histopathological analyses. Regardless, the present study investigated the transient local direct effects of SGLT2-inhibitors on the cardiac tissue during the acute phase of IRI; our findings therefore do not conflict with the consistent cardioprotective results from other in vivo experimental studies [44, 49–56] or clinical trials [31–33], all of which investigated the systemic effects of SGLT2-inhibitors over a longer period. Furthermore, the present findings suggest that potential mechanisms underlying the cardiovascular benefits of SGLT2-inhibitors may depend largely on the systemic inter-organ network, including cardiorenal syndrome [57–59].

GLUT1 is another major glucose transporter in the heart that is largely responsible for the basal cardiac glucose transport in an insulin-independent manner [6, 10]. Previous studies have shown that GLUT1 also exerts important cardioprotective actions, mainly in chronic pathological settings [15, 60]. Since GLUT1 is a facilitated energy-independent transporter that usually localizes to the plasma membrane, its expression at the plasma membrane is considered to directly reflect its activity. Given that we did not detect any significant change in the GLUT1 expression at the plasma membrane, regardless of the diet condition, GLUT1 may have a limited impact, at least in the present experimental model.

Some studies suggested that 2-DG might be a relatively poor substrate for SGLT1, although this glucose analog has been used for the glucose uptake assay of SGLTs in previous studies [7, 19, 61]. Thus, we cannot completely deny the possibility that impairment of cardiac glucose uptake by phlorizin may be due, at least in part, to the non-specific inhibition of other glucose transporters. However, a series of previous studies using phlorizin indicated that phlorizin does inhibit cardiac SGLT1 [25, 62–64]. Lambert et al. showed that 200 µM of phlorizin reduced the myocardial intracellular sodium concentration, which rules out any involvement in GLUTs (namely, sodium-independent glucose transporters) [21]. Furthermore, a relatively low dose of phlorizin was used in the present study compared to that used in the previous studies (200–10 mM) [21, 25, 39, 62, 63], thus minimizing the non-specific inhibitory effects of phlorizin on other glucose transporters. Finally, it is highly possible that phlorizin-perfusion specifically inhibits cardiac SGLT1 in the current ex vivo Langendorff model, since non-specific inhibitory effects on glucose transporters are observed only when phlorizin is broken down in the small intestine to its aglycone form, phloretin [65, 66]. In fact, the plasma membrane expression patterns of GLUT4 and GLUT1 were unchanged by phlorizin administration at all points of IRI under both diet conditions (Additional file 1: Fig. S4). Likewise, previous studies by other groups [22, 24] showed that there was no significant differences in the levels of membrane-associated GLUT1 or GLUT4 in mice with cardiomyocyte-specific knockdown of SGLT1, suggesting that the expression/activity of SGLT1 and GLUTs may be regulated independently, without any mutual interaction in the heart.

It is also important to assess the expression of those glucose transporters in isolated cardiomyocytes, considering that not only myocardial SGLT1 and other glucose transporters but also those residing in cardiac fibroblasts [40] and endothelial cells [67] may play a substantial role under diverse pathological conditions, such as ischemic heart diseases as well as diabetes (insulin resistant conditions), and the regulation of the expression of those glucose transporters might differ among cell types.

In this context, it would be interesting to clarify the effects of SGLT1 inhibition during IRI in vivo in order to examine its influence on the systemic inter-organ network as well as macrophages and other blood cells circulating coronary arteries. On the other hand, the Langendorff system is useful for evaluating the direct local effects of glucose transporters in the heart more clearly, as this system is not associated with changes in systemic substrate metabolism or neurohumoral factors activated under pathological conditions, such as diabetes.

Given these limitations associated with the present study, future investigations should conduct a series of experiments using a conditional (transient) cardiomyocyte-specific SGLT1 knockout model (rather than permanent knockdown from birth) in order to clarify the complete in vivo characterization of the relatively acute actions of SGLT1 in the cardiomyocytes.

## Conclusions

We determined that the impairment of the cardiac functional recovery and increased myocardial injury associated with the reduction in the myocardial glucose uptake by phlorizin during IRI were more pronounced in diet-induced obese mice hearts than in the hearts of mice fed a normal diet, while no harmful effects induced by any SGLT2-inhibitors were observed in the same setting. Given that SGLT1 and GLUTs function independently without any mutual interaction, at least under the conditions in the present study, there is an increasing reliance on SGLT1 for cardiac functional recovery after ischemia–reperfusion via enhanced myocardial glucose utilization, particularly under insulin-resistant conditions, in which GLUT4 upregulation in response to IRI is compromised.

## Additional file

**Additional file 1: Table S1.** Baseline cardiac function of perfused hearts with or without SGLT2-inhibitors perfusion in HFD-fed mice. **Table S2.** Baseline cardiac function of perfused hearts with or without tofogliflozin perfusion in normal chow-fed mice. **Fig. S1.** The profiles of other hemodynamic parameters. The profiles of RPP (A), LVEDP (B), and positive and negative dp/dt (C) measured at the indicated time points during ischemia–reperfusion in NFD (open black square; n = 6), NFD phlorizin-perfused (filled black square; n = 6), HFD (open pink square; n = 8), and HFD phlorizin-perfused (filled pink square; n = 8) hearts are shown. *P < 0.05 and **P < 0.01 versus NFD group at each time point; ^†^P < 0.05 and ^‡^P < 0.01 versus HFD group at each time point. min, minutes; RPP, rate pressure product; LVEDP, left ventricular end-diastolic pressure. **Fig. S2.** SGLT2 mRNA was not detected in the heart from both NFD and HFD mice. The quantitative reverse transcription polymerase chain reaction (QRT-PCR) data indicating the SGLT2 gene expression levels in the hearts from either NFD (A) or HFD (B), or in the mouse intestine as the negative control and in the kidney as the positive control (C) (n = 3 each). (D) The QRT-PCR data were normalized to GAPDH. The data are shown as the fold change normalized to the levels found in the kidney (C). **Fig. S3.** Expression of GLUT1 in the murine hearts during ischemia–reperfusion. Representative immunoblots of GLUT1 in the plasma membrane fraction from the murine perfused hearts at baseline period measured at the end of the 10-minute pre-ischemia perfusion (A), and before and after IRI (B) are shown. (C) Densitometric quantitation normalized to the level of GLUT1 expression in NFD hearts before IRI is shown (NFD or HFD without IRI; n = 5 each, with IRI; n = 3 each). In both (A) and (B), immunoblots of Na^+^/K^+^ ATPase from the same membrane are shown as a loading control for the membrane fraction. **Fig. S4.** Expression of GLUT4, SGLT1 and GLUT1 in murine hearts during ischemia–reperfusion with or without phlorizin-perfusion. Representative immunoblots of GLUT4, SGLT1 and GLUT1 in the plasma membrane fraction from the murine perfused hearts before and after IRI with or without phlorizin-perfuion (A) are shown. The immunoblot of Na^+^/K^+^ ATPase from the same membrane are shown as a loading control for the membrane fraction. (B) Densitometric quantitation normalized to the level of either GLUT4, SGLT1 or GLUT1 expression in NFD hearts before IRI are shown (n = 3 each). *P < 0.05, **P < 0.01 versus NFD hearts without phlorizin perfusion before IRI; ^#^P < 0.05 versus NFD hearts with phlorizin perfusion before IRI; ^‡^P < 0.01 versus NFD hearts without phlorizin perfusion after IRI; ^§^P < 0.05 versus NFD hearts with phlorizin perfusion after IRI.

## Data Availability

The datasets used and/or analysed during the current study are available from the corresponding author on reasonable request.

## Abbreviations

2-DG: 2-deoxy-d-glucose
AAR: area at risk
AMPK: AMP-activated protein kinase
ATP: adenosine triphosphate
AUC: area under the curve
Cana: canagliflozin
CPK: creatine phosphokinase
DMSO: dimethylsulfoxide
+dp/dt_max_: maximum rate of contraction
−dp/dt_min_: maximum rate of relaxation
GAPDH: glyceraldehyde 3-phosphate dehydrogenase
GLUT: facilitated glucose transporter
HFD: high-fat diet
HIF-1α: hypoxia-inducible factor 1α
HR: heart rate
I: ischemia
Ipra: ipragliflozin
IRI: ischemia–reperfusion injury
LVDP: left ventricular developed pressure
LVEDP: left ventricular end-diastolic pressure
LVSP: left ventricular systolic pressure
min: minutes
NFD: normal-fat diet
QRT-PCR: quantitative reverse transcription polymerase chain reaction
R: reperfusion
RNA: ribonucleic acid
RPP: rate pressure product
SGLT: sodium–glucose cotransporter
SGLT2i: SGLT2-inhibitor
Tofo: tofogliflozin
TTC: triphenyltetrazolium chloride
