# Supplementary material for: Cardiac ischemia–reperfusion injury under insulin-resistant conditions: SGLT1 but not SGLT2 plays a compensatory protective role in diet-induced obesity
Source: Cardiovasc Diabetol. 2019 Jul 1;18:85. doi: 10.1186/s12933-019-0889-y (PMC6604374; doi:10.1186/s12933-019-0889-y)
Supplement: Supplementary file 1 — Additional file 1: Table S1. Baseline cardiac function of perfused hearts with or without SGLT2-inhibitors perfusion in HFD-fed mice. Table S2. Baseline cardiac function of perfused hearts with or without tofogliflozin perfusion in normal chow-fed mice. Fig. S1. The profiles of other hemodynamic parameters. The profiles of RPP (A), LVEDP (B), and positive and negative dp/dt (C) measured at the indicated time points during ischemia–reperfusion in NFD (open black square; n = 6), NFD phlorizin-perfused (filled black square; n = 6), HFD (open pink square; n = 8), and HFD phlorizin-perfused (filled pink square; n = 8) hearts are shown. *P < 0.05 and **P < 0.01 versus NFD group at each time point; †P < 0.05 and ‡P < 0.01 versus HFD group at each time point. min, minutes; RPP, rate pressure product; LVEDP, left ventricular end-diastolic pressure. Fig. S2. SGLT2 mRNA was not detected in the heart from both NFD and HFD mice. The quantitative reverse transcription polymerase chain reaction (QRT-PCR) data indicating the SGLT2 gene expression levels in the hearts from either NFD (A) or HFD (B), or in the mouse intestine as the negative control and in the kidney as the positive control (C) (n = 3 each). (D) The QRT-PCR data were normalized to GAPDH. The data are shown as the fold change normalized to the levels found in the kidney (C). Fig. S3. Expression of GLUT1 in the murine hearts during ischemia–reperfusion. Representative immunoblots of GLUT1 in the plasma membrane fraction from the murine perfused hearts at baseline period measured at the end of the 10-minute pre-ischemia perfusion (A), and before and after IRI (B) are shown. (C) Densitometric quantitation normalized to the level of GLUT1 expression in NFD hearts before IRI is shown (NFD or HFD without IRI; n = 5 each, with IRI; n = 3 each). In both (A) and (B), immunoblots of Na+/K+ ATPase from the same membrane are shown as a loading control for the membrane fraction. Fig. S4. Expression of GLUT4, SGLT1 and GLUT1 in mur [file 12933_2019_889_MOESM1_ESM.docx]

**Additional Files**

**Cardiac ischemia-reperfusion injury under insulin-resistant conditions: SGLT1 but not SGLT2 plays a compensatory protective role in diet-induced obesity**

Akira Yoshii (yoshii_no_akira@yahoo.co.jp), Tomohisa Nagoshi (tnagoshi@jikei.ac.jp), Yusuke Kashiwagi (kashiwa2211@gmail.com), Haruka Kimura (kimuraha@jikei.ac.jp), Yoshiro Tanaka (tanakayoshiro11@gmail.com), Yuhei Oi (yuhei_bj@hotmail.com), Keiichi Ito (keke_ito@yahoo.co.jp), Takuya Yoshino (t.y.999@hotmail.co.jp), Toshikazu D. Tanaka (jjuichi@gmail.com), and Michihiro Yoshimura (m.yoshimura@jikei.ac.jp)

Division of Cardiology, Department of Internal Medicine,

The Jikei University School of Medicine

3-25-8，Nishi-Shinbashi, Minato-ku, Tokyo, 105-8461, JAPAN

Tel: +81-3-3433-1111 (ex.3261), Fax: +81-3-3459-6043

**Correspondence to:** Tomohisa Nagoshi, M.D.,Ph.D.

E-mail: [tnagoshi@jikei.ac.jp](mailto:tnagoshi@jikei.ac.jp)

**Table S1** Baseline cardiac function of perfused hearts with or without SGLT2-inhibitors perfusion in HFD-fed mice.

|  | Tofogliflozin  (n=5) | Ipragliflozin  (n=6) | Canagliflozin (n=8) |
| --- | --- | --- | --- |
| LVSP, mmHg | 133±6.6 | 139±8.7 | 130±9.0 |
| LVEDP, mmHg | 8.9±0.3 | 9.1±0.2 | 8.7±0.4 |
| LVDP, mmHg | 124±6.8 | 130±8.8 | 122±8.8 |
| +dp/dt, mmHg/s | 4127±270* | 4481±524 | 3993±246 |
| -dp/dt, mmHg/s | -2960±312 | -2825±236 | -3232±192 |
| HR, bpm | 281±22 | 232±38 | 307±39 |
| RPP, mmHg・bpm | 35189±3706 | 29641±4384 | 36041±4453 |
| Coronary flow, ml/min | 3.41±0.85 | 3.10±0.29 | 4.28±0.42 |

*P<0.05 versus HFD group.

LVSP, left ventricular systolic pressure; LVEDP, left ventricular end-diastolic pressure; LVDP, left ventricular developed pressure; HR, heart rate; RPP, rate pressure product.

**Table S2** Baseline cardiac function of perfused hearts with or without tofogliflozin perfusion in normal chow-fed mice.

|  | Control  (n=10) | Tofogliflozin 5 µM  (n=8) | Tofogliflozin 50 µM  (n=8) |
| --- | --- | --- | --- |
| LVSP, mmHg | 101±6.8 | 93.1±5.7 | 88.9±3.6 |
| LVEDP, mmHg | 8.5±0.6 | 8.6±0.5 | 9.7±0.5 |
| LVDP, mmHg | 92.7±7.2 | 84.5±5.6 | 79.2±3.8 |
| +dp/dt, mmHg/s | 3063±331 | 2567±342 | 2283±157 |
| -dp/dt, mmHg/s | -2406±183 | -2260±143 | -2416±147 |
| HR, bpm | 367±17 | 345±13 | 366±16 |
| RPP, mmHg・bpm | 33877±2696 | 29324±2541 | 28103±2282 |
| Coronary flow, ml/min | 2.85±0.28 | 2.58±0.17 | 3.54±0.32 |

LVSP, left ventricular systolic pressure; LVEDP, left ventricular end-diastolic pressure; LVDP, left ventricular developed pressure; HR, heart rate; RPP, rate pressure product.

**
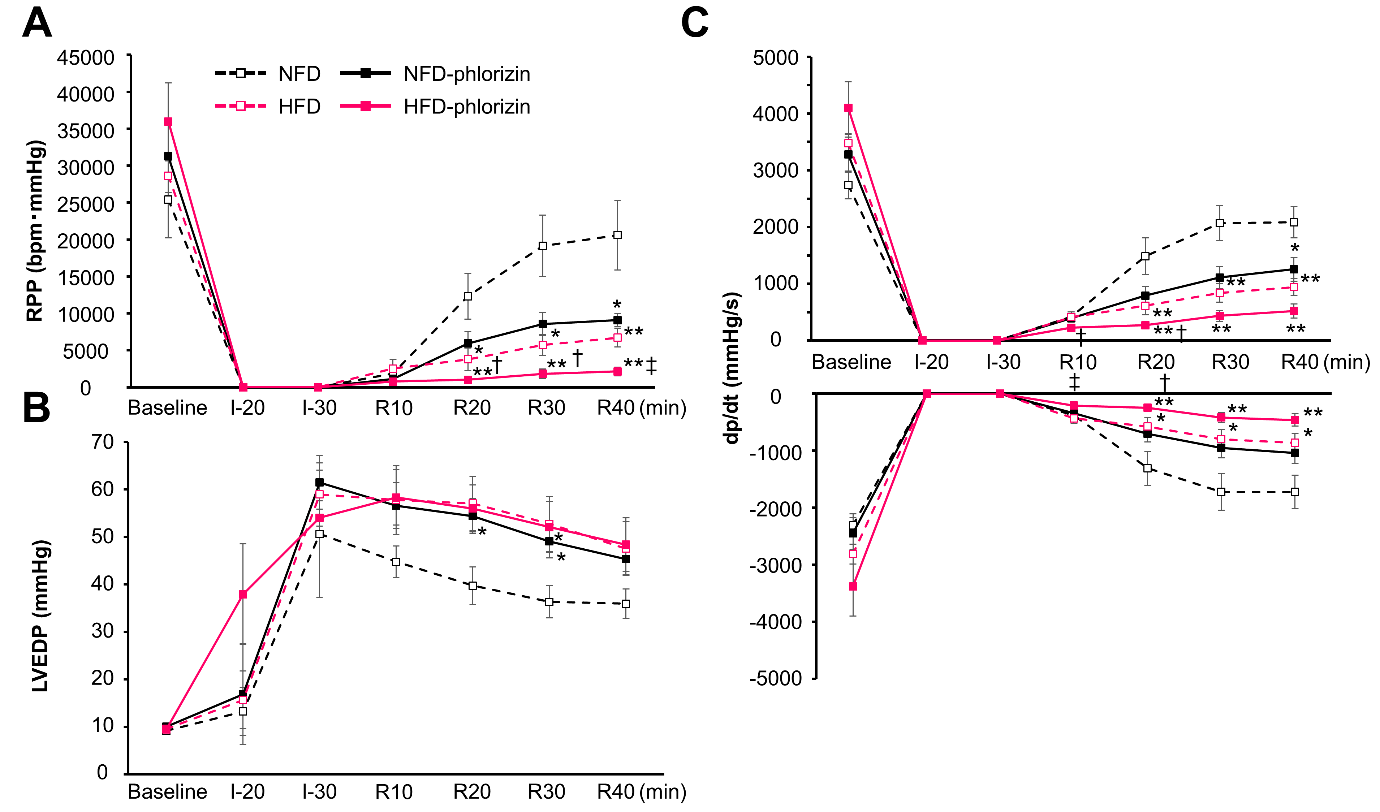
**

**Fig. S1 The profiles of other hemodynamic parameters.** The profiles of RPP **(A)**, LVEDP **(B)**, and positive and negative dp/dt **(C)** measured at the indicated time points during ischemia-reperfusion in NFD (open black square; n=6), NFD phlorizin-perfused (filled black square; n=6), HFD (open pink square; n=8), and HFD phlorizin-perfused (filled pink square; n=8) hearts are shown. *P<0.05 and **P<0.01 versus NFD group at each time point; ^†^P<0.05 and ^‡^P<0.01 versus HFD group at each time point.

min, minutes; RPP, rate pressure product; LVEDP, left ventricular end-diastolic pressure.

**
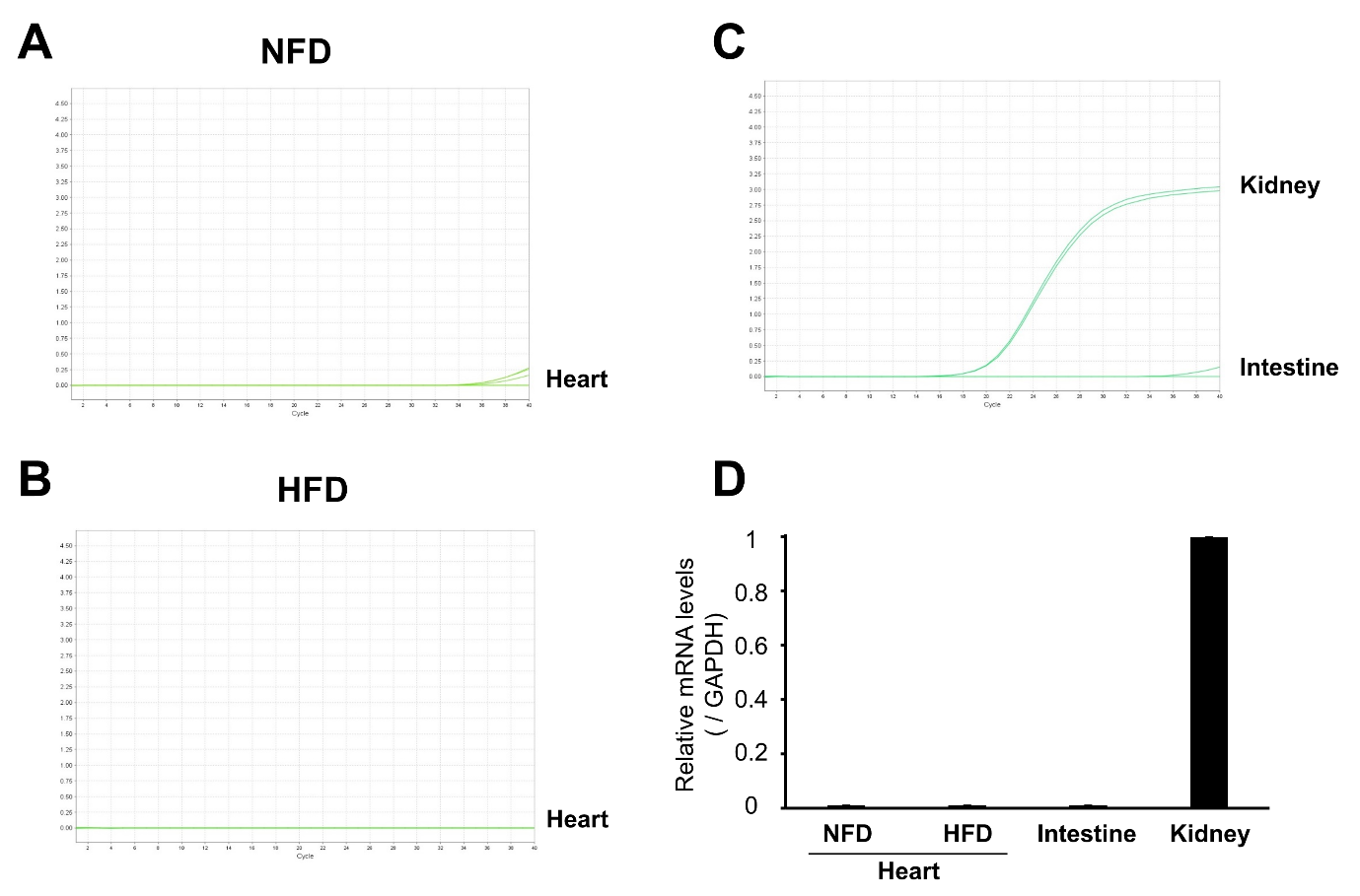
**

**Fig. S2 SGLT2 mRNA was not detected in the heart from both NFD and HFD mice.** The quantitative reverse transcription polymerase chain reaction (QRT-PCR) data indicating the SGLT2 gene expression levels in the hearts from either NFD **(A)** or HFD **(B)**, or in the mouse intestine as the negative control and in the kidney as the positive control **(C)** (n=3 each). **(D)** The QRT-PCR data were normalized to GAPDH. The data are shown as the fold change normalized to the levels found in the kidney (C).


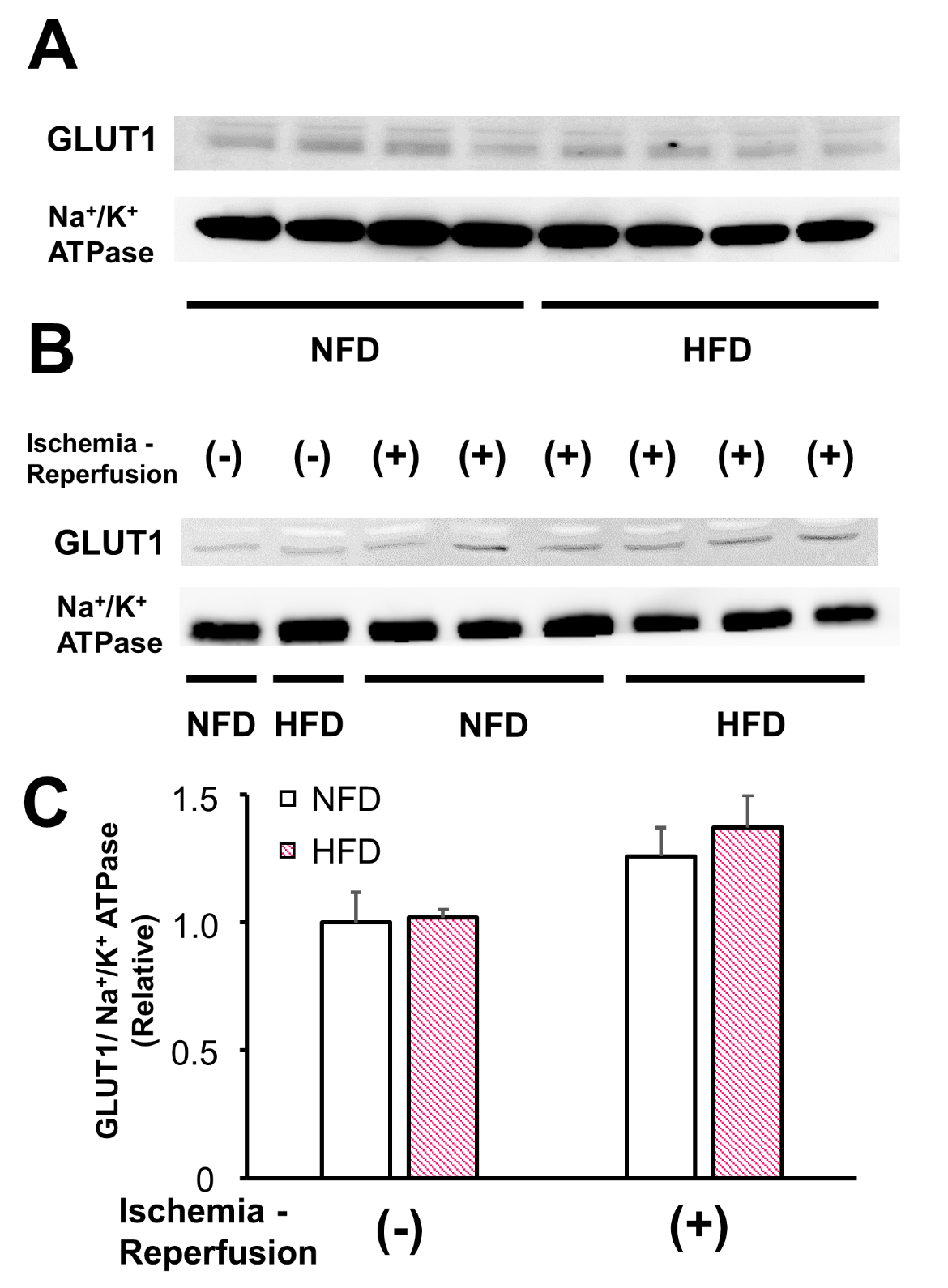


**Fig. S3 Expression of GLUT1 in the murine hearts during ischemia-reperfusion.** Representative immunoblots of GLUT1 in the plasma membrane fraction from the murine perfused hearts at baseline period measured at the end of the 10-minute pre-ischemia perfusion **(A)**, and before and after IRI **(B)** are shown. **(C)** Densitometric quantitation normalized to the level of GLUT1 expression in NFD hearts before IRI is shown (NFD or HFD without IRI; n=5 each, with IRI; n=3 each). In both (A) and (B), immunoblots of Na^+^/K^+^ ATPase from the same membrane are shown as a loading control for the membrane fraction.

**
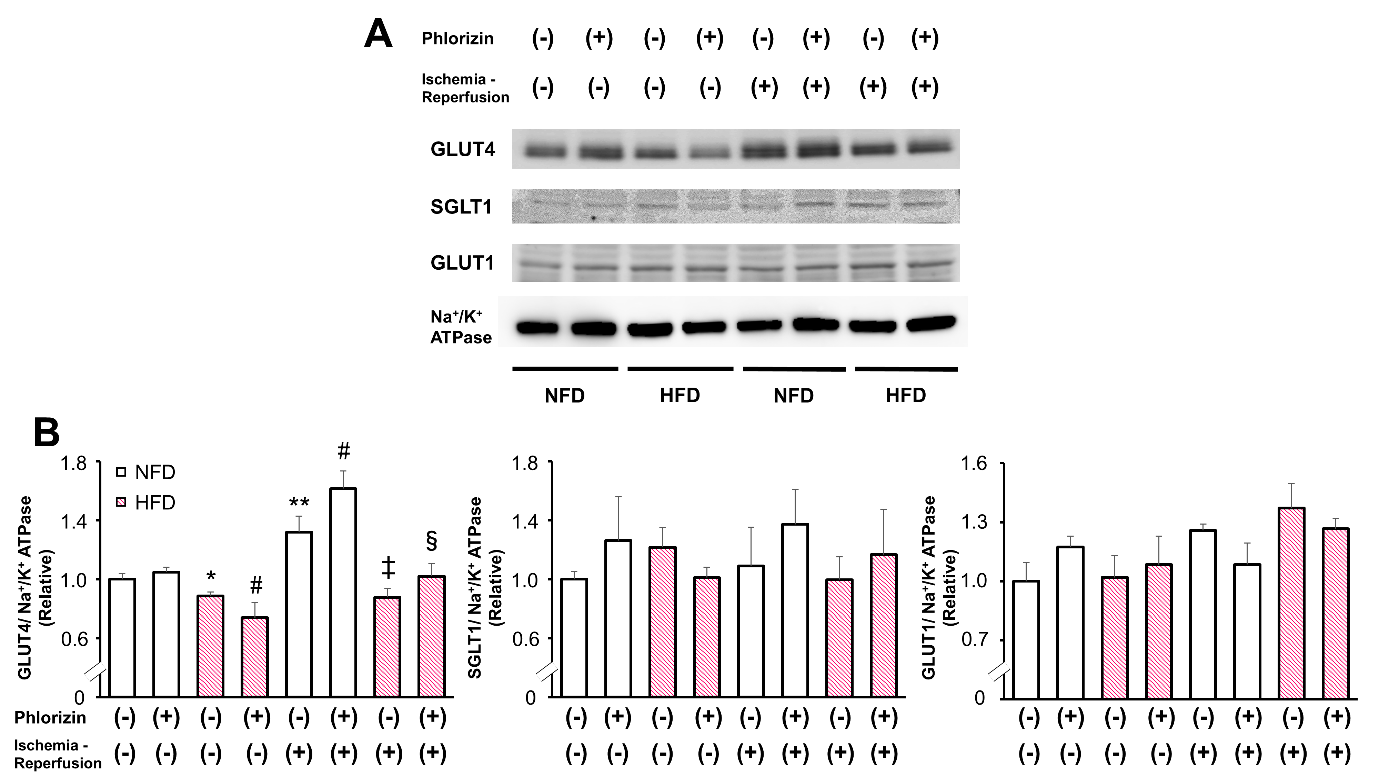
**

**Fig. S4 Expression of GLUT4, SGLT1 and GLUT1 in murine hearts during ischemia-reperfusion with or without phlorizin-perfusion.** Representative immunoblots of GLUT4, SGLT1 and GLUT1 in the plasma membrane fraction from the murine perfused hearts before and after IRI with or without phlorizin-perfuion **(A)** are shown. The immunoblot of Na^+^/K^+^ ATPase from the same membrane are shown as a loading control for the membrane fraction. **(B)** Densitometric quantitation normalized to the level of either GLUT4, SGLT1 or GLUT1 expression in NFD hearts before IRI are shown (n=3 each). *P<0.05, **P<0.01 versus NFD hearts without phlorizin perfusion before IRI; ^#^P<0.05 versus NFD hearts with phlorizin perfusion before IRI; ^‡^P<0.01 versus NFD hearts without phlorizin perfusion after IRI; ^§^P<0.05 versus NFD hearts with phlorizin perfusion after IRI.
